# Ventriculo-arterial coupling for predicting cardiac index increase in infants after heart surgery

**DOI:** 10.1093/icvts/ivad064

**Published:** 2023-05-09

**Authors:** Wenjuan Li, Yongxuan Peng, Zhihao Li, Jihong Huang

**Affiliations:** Department of Pediatric Cardiology, Xinhua Hospital, Affiliated to Shanghai Jiao Tong University School of Medicine, Shanghai, China; Department of Pediatric Cardiology, Xinhua Hospital, Affiliated to Shanghai Jiao Tong University School of Medicine, Shanghai, China; Department of Pediatric Cardiovascular and Thoracic Surgery, Shanghai Children's Medical Center, Medical School of Shanghai Jiaotong University, Shanghai, China; Department of Pediatric Cardiology, Xinhua Hospital, Affiliated to Shanghai Jiao Tong University School of Medicine, Shanghai, China

**Keywords:** Milrinone, Paediatric, Cardiac intensive care, Congenital heart surgery, Ventriculo-arterial coupling

## Abstract

**OBJECTIVES:**

The aim of this study was to test the ability of ventriculo-arterial coupling (VAC) to predict cardiac index (CI) response after milrinone infusion.

**METHODS:**

This was a retrospective, observational study. We measured arterial blood pressure and echocardiography-derived variables, including CI, systemic vascular resistance index, arterial elastance (Ea) and end-systolic ventricular elastance before and after 18–24 h of milrinone infusion. VAC was calculated as the ratio of Ea to end-systolic elastance. Infants with over 15% increase in the CI were defined as CI responders. Logistical regression was used to evaluate predictors of CI responders.

**RESULTS:**

We enrolled 92 infants who underwent cardiac surgery and received milrinone infusion, of whom 45 infants were CI responders. High VAC (odds ratio = 5.534, 95% confidence interval 2.339–13.090) and high Ea (odds ratio = 3.035, 95% confidence interval 1.459–6.310) were independently associated with cardiac index responders. Pre-milrinone VAC predicted CI responsiveness with a cut-off value of 1.12 (area under the curve = 0.900, 95% confidence interval 0.819–0.953, *P* < 0.0001). Furthermore, we observed a decrease in the infant's VAC, Ea and systemic vascular resistance index after milrinone infusion.

**CONCLUSIONS:**

In infants with congenital heart disease after surgery, a pre-milrinone VAC >1.12 can predict the increase in the CI following milrinone infusion.

**Figure:**
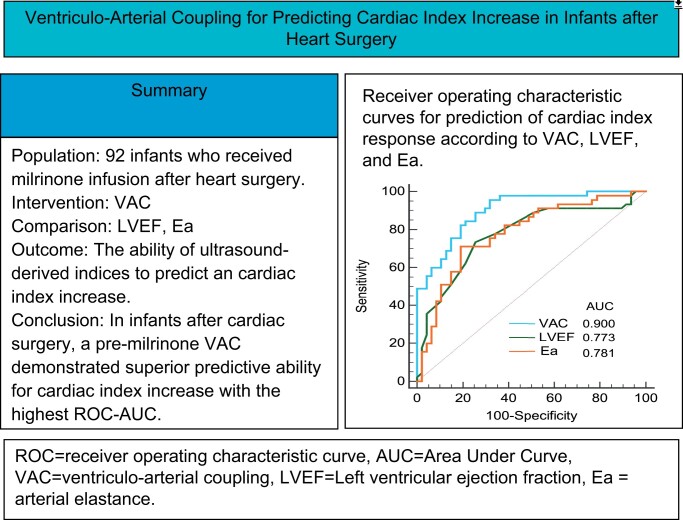

## INTRODUCTION

Children with congenital heart disease (CHD) often require heart surgery at a young age. They are at risk for postoperative low cardiac output syndrome (LCOS). Cardiopulmonary bypass (CPB) is an essential technique used during cardiac surgery, but it can result in significant myocardial damage. Oh *et al.* [1] demonstrated that high ventriculo-arterial coupling (VAC) after CPB was associated with worse early postoperative outcomes. VAC is calculated as the ratio of arterial elastance (Ea) to left ventricular end-systolic elastance (Ees). The concept of VAC arises from the logical connection between the heart and the arterial system, which are anatomically and functionally linked. High VAC levels suggest an inefficient relationship between the heart and arterial system, either due to inadequate ventricular contraction or high arterial impedance. VAC has been widely used in cardiology to investigate underlying physiology for many years [2, 3]. Recently, there has been an increase in the number of clinical studies focusing on VAC in infants with congenital heart surgery or low cardiac output syndrome [1, 4, 5]. Milrinone infusion is widely used to reduce the incidence of LCOS [6, 7]. However, there is insufficient evidence to support the effectiveness of milrinone in preventing LCOS in post-cardiac surgery infants compared to a placebo [8]. Milrinone treatment is mainly based on clinician preference. At the bedside, predicting the haemodynamic effects of milrinone is challenging. Oh *et al.* demonstrated that a VAC of >1.49 could predict the use of milrinone in infants after cardiac surgery [1]. This study aimed to evaluate the predictive ability of VAC for cardiac index (CI) improvement following milrinone infusion in post-cardiac surgery infants.

## MATERIALS AND METHODS

### Patients

This was a two-centre retrospective, observational trial. The study was performed at Shanghai Children's Medical Center and Shanghai Xinhua Hospital.

### Ethics statement

Institutional Review Board approval has been obtained from both the Shanghai Children's Medical Center (approval number: SCMCIRB-K2018053) and the Shanghai Xinhua Hospital (approval number: XHEC-QT-2021-042). Informed consent was waived.

Between April 2019 and December 2021, we conducted a retrospective analysis of CHD infants who underwent cardiac surgery and received milrinone infusion (maintained at 0.25–0.75 μg/kg/min) upon arrival to the ICU. Routine echocardiography was performed twice on the infants (before milrinone infusion and on postoperative day 1). Milrinone was indicated for congestive cardiac failure and postoperative cardiac surgery. Contraindications to milrinone included severe obstructive aortic valvular disease or hypertrophic subaortic stenosis, renal dysfunction and hypotension.

Inclusion criteria were as follows: (i) milrinone infusion was initiated within the first 6 h after cardiac surgery and continued for ≥18 h; (ii) body weight ≥2.5 kg; and (iii) preoperative left ventricular ejection fraction (LVEF) ≥50%. Exclusion criteria included (i) palliation of single-ventricle physiology; (ii) administration of any vasodilator (such as dobutamine) or changes to the maintenance infusion rate of vasoactive drugs during the study; (iii) inadequate postoperative ultrasound image quality; (iv) residual major cardiac lesions with a residual lesion score class = 3 [9]; (v) insufficient blood volume, which was defined as cases where bedside echocardiography shows >20% variation in inferior vena cava before milrinone infusion; and (vi) changes to ventilator settings during the study period.

The patients received routine ICU care and were continuously monitored for vital signs. All patients were sedated using dexmedetomidine (initial dose 0.5 μg/kg/h) and midazolam (initial dose 2 μg/kg/min), and the sedatives were adjusted based on the sedation score. Throughout the study, the patients were mechanically ventilated using volume-controlled mode with a tidal volume of 7–9 ml/kg per body weight and positive end-expiratory pressure of 3–5 cmH_2_O.

### Clinical and haemodynamic variables

We collected demographic data for each patient, including age, sex, body weight, diagnosis, body surface area (BSA), history of prematurity, CPB time, aortic cross-clamp time and vasoactive-inotropic score. Vasoactive-inotropic score was calculated following a previous report [10]. Clinical and haemodynamic data were also collected, including heart rate, systolic blood pressure, diastolic blood pressure, mean blood pressure and central venous pressure (CVP), both before milrinone infusion and on postoperative day 1.

Twice echocardiography assessments were conducted both before and 18–24 h after milrinone infusion. All echocardiogram examinations were performed by 2 cardiologists who reached a consensus and who were blind to corresponding clinical information. The measurement of the diameter of inferior vena cava diameter and abdominal aorta, LVEF, pre-ejection and total ejection times, left ventricular outflow tract diameter and velocity time integral was conducted according to the method outlined by Trambaiolo *et al.* [11]. All parameters were measured over 5 consecutive cardiac cycles and averaged. Echocardiography-derived haemodynamic variables, such as LV stroke volume, stroke volume index (SVI), CI and systemic vascular resistance index (SVRI), are presented in Supplementary Material 1.

Infants who exhibited a CI increase of over 15% were categorized into the CI response group, while the remaining infants were classified into the CI non-response group.

### Left ventricular end-systolic elastance, arterial elastance and ventriculo-arterial coupling

Ees, Ea and VAC were obtained using the iElastance© application on Apple iOS devices. This application functions as a bedside calculator utilizing the single-beat technique of Chen's algorithm [12]. To normalize Ees and Ea, BSA measurements were referenced to previous literature [13].

### Statistical analysis

A sample size of 48 (out of 92 subjects included in our study) was calculated to be sufficient to demonstrate that VAC predicts an increase in CI with an area under the curve (AUC) of >0.8 and a power of 90%, at a significance level of 0.05. Data are expressed as proportions (%), means (standard deviation) or medians (interquartile range) as appropriate. Infants were classified based on the magnitude of CI increase. The nonparametric Wilcoxon rank-sum test, Student’s *t*-test, paired *t*-tests and the Mann–Whitney *U*-test were utilized to assess statistical significance. Univariable and multivariable logistic regression analyses were conducted to evaluate pre-milrinone variables and their association with CI responders. The odds ratio (OR) was reported with 95% confidence intervals. Receiver operating characteristic (ROC) curves were established for VAC, Ea and LVEF to predict a >15% increase in CI, and the curves were compared using the DeLong test. A *P*-value of <0.05 was set as the threshold for statistical significance. Statistical analysis was performed using R Studio1.4.

## RESULTS

**Figure 1:**
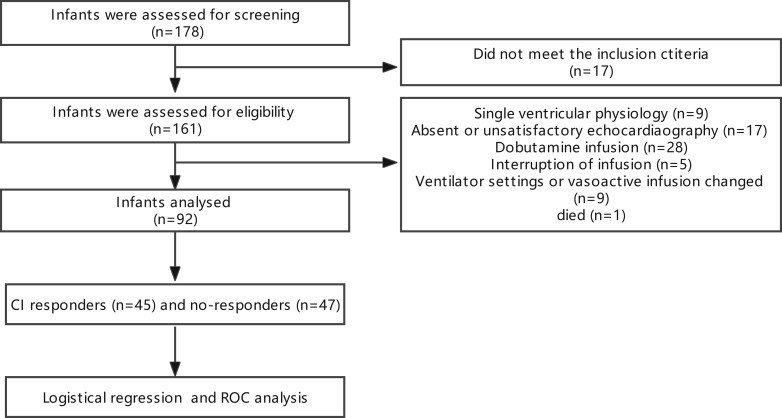
Study flowchart.

A total of 161 infants underwent CPB surgery, of whom 69 infants were excluded. The study was based on 92 infants who had undergone cardiac surgery (Fig. 1).

Table 1 presents the demographic characteristics of the subjects. Of the total, 39 patients were female (42.4%). The mean age and weight were 128 days and 5.26 kg, respectively. Out of all subjects, 45 were CI responders (48.91%), while 47 were non-responders (51.09%). Further details regarding patient diagnosis are provided in Supplementary Material 2.

**Table 1:** Characteristics of the study participants

Variables	*N* = 92
Age (days)	128 (101)
Female	39 (42.4%)
Body weight (kg)	5.26 (1.62)
RACHS-1	
1–2	52 (56.5%)
3	29 (31.5%)
4	9 (9.8%)
5–6	2 (2.2%)
Bypass time (min)	80 (35)
Pre-milrinone lactate (mmol/l)	2.7 (1.7, 4.8)
Pre-milrinone VIS	5.5 (4.8)

Data are expressed as *n* (%), the mean (standard deviation) or the median with the interquartile range.

RACHS-1: Risk Adjustment in Congenital Heart Surgery-1 score; VIS: vasoactive-inotropic score.

### The changes in haemodynamic variables following milrinone infusion

Table 2 shows that prior to milrinone infusion, CI responders had higher pre-milrinone values for SVRI, Ea and VAC, while their LVEF and SVI were lower compared to CI non-responders. Following 18–24 h of milrinone infusion, significant decreases in SVRI, Ea and VAC were observed in the CI responders, along with increases in LVEF and SVI. However, there was no significant change in SVRI, Ea or VAC before and after milrinone infusion in non-responders (Table 2).

**Table 2:** Comparison of haemodynamic variables before and after milrinone infusion

Haemodynamic variables	Pre-milrinone	Post-milrinone	*P*-Value
LVEF (%)			
All (*n* = 92)	51.8 (6.5)	55.2 (5.3)	<0.001
CI responders (*n* = 45)	47.5 (7.1)	55.0 (5.5)	<0.001
CI non-responders (*n* = 47)	53.4 (7.5)^a^	55.9 (5.2)	0.063
SVRI (dynes* s *m^2^/cm^5^)			
All (*n* = 92)	1931 (774)	1599 (536)	<0.001
CI responders (*n* = 45)	2064 (784)	1520 (472)	<0.001
CI non-responders (*n* = 47)	1720 (528)^a^	1616 (672)	0.407
SVI (ml/m^2^)			
All (*n* = 92)	21.4 (5.1)	23.2 (6.2)	0.033
CI responders (*n* = 45)	18.8 (3.5)	22.4 (4.1)	<0.001
CI non-responders (*n* = 47)	23.6 (4.9)^a^	23.2 (7.1)	0.751
CI (l/min/m^2^)			
All (*n* = 92)	2.89 (0.52)	3.22 (0.53)	<0.0001
CI responders (*n* = 45)	2.63 (0.35)	3.17 (0.45)	<0.0001
CI non-responders (*n* = 47)	3.14 (0.56)^a^	3.24 (0.66)	0.430
CVP (mmHg)			
All (*n* = 92)	7.7 (3.1)	8.0 (2.9)	0.473
CI responders (*n* = 45)	7.7 (3.3)	8.0 (2.8)	0.630
CI non-responders (*n* = 47)	7.7 (3.2)	7.9 (3.0)	0.752
Ea (mmHg/ml/m^2^)			
All (*n* = 92)	4.17 (1.32)	3.51 (0.92)	<0.001
CI responders (*n* = 45)	4.70 (1.15)	3.47 (0.74)	<0.001
CI non-responders (*n* = 47)	3.66 (0.84)^a^	3.54 (1.03)	0.537
Ees (mmHg/ml/m^2^)			
All (*n* = 92)	3.46 (1.05)	3.47 (0.51)	0.682
CI responders (*n* = 45)	3.45 (1.15)	3.43 (0.34)	0.911
CI non-responders (*n* = 47)	3.47 (0.87)	3.50 (0.54)	0.841
VAC			
All (*n* = 92)	1.25 (0.28)	1.00 (0.18)	<0.001
CI responders (*n* = 45)	1.43 (0.29)	1.01 (0.20)	<0.001
CI non-responders (*n* = 47)	1.07 (0.17)^a^	1.00 (0.18)	0.059

Data are expressed as the mean (standard deviation). *P*-value refers to the comparison between post- and pre-milrinone.

a *P* < 0.05 refers to a comparison between CI responders and non-responders.

CI: cardiac index; Ea: arterial elastance index; Ees: left ventricular end-systolic elastance; LVEF: left ventricular ejection fraction; SVI: stroke volume index; SVRI: systemic vascular resistance index; VAC: ventriculo-arterial coupling,.

After 18–24 h of milrinone infusion in CI responders, there was a significant decrease in Ea from 4.70 (1.15) to 3.47 (0.74) mmHg/l/m^2^ (*P* < 0.001). However, there was no significant change observed in Ees. As a result, there was a significant improvement in VAC from 1.43 (0.29) to 1.01 (0.20) (*P* < 0.001), along with a significant increase in SVI from 18.8 (3.5) to 22.4 (4.1) ml/m^2^.

A more detailed description of haemodynamic variables is given in Supplementary Material 3.

### Logistic regression analysis

Univariable binomial logistic regression revealed that 5 variables were significantly associated with CI responders. (Table 3): LVEF had an OR of 0.866 (95% confidence interval 0.795–0.943, *P* < 0.001), SVRI had an OR of 1.001 (95% confidence interval 1.000–1.001, *P* = 0.017), SVI had an OR of 0.784 (95% confidence interval 0.694–0.885, *P* < 0.001), Ea had an OR of 2.578 (95% confidence interval 1.620–4.102, *P* < 0.001) and VAC had an OR of 2.697 (95% confidence interval 1.757–4.139, *P* < 0.001).

**Table 3:** Univariable logistic regressions with characteristics and haemodynamic covariates associated with cardiac index increase

	OR	95% confidence interval	*P*-Value
Age	0.998	0.994–1.002	0.334
Sex	1.179	0.515–2.698	0.679
Weight	0.963	0.747–1.240	0.768
RACHS-1	1.043	0.689–1.582	0.844
Bypass time	1.004	0.992–1.016	0.485
Lactate	1.000	0.924–1.081	0.994
VIS	0.971	0.897–1.052	0.472
LVEF	0.866	0.795–0.943	0.001
SVRI	1.001	1.000–1.001	0.017
SVI	0.784	0.694–0.885	0.000
Ea × 10	2.578	1.620–4.102	0.000
Ees	0.928	0.633–1.360	0.702
VAC × 10	2.697	1.757–4.139	0.000
CVP	1.038	0.894–1.204	0.625
Heart rate	1.017	0.998–1.036	0.159
SBP	1.008	0.989–1.028	0.407

CI: cardiac index; CVP: central venous pressure; Ea: arterial elastance index; Ees: left ventricular end-systolic elastance; LVEF: left ventricular ejection fraction; OR: odds ratio; RACHS-1: Risk Adjustment in Congenital Heart Surgery-1 score; SBP: systolic blood pressure; SVI: stroke volume index; SVRI: systemic vascular resistance index; VAC: ventriculo-arterial coupling; VIS: vasoactive-inotropic score.

Univariable predictors with a *P*-value of <0.10 were selected for inclusion in a multivariable model. In the multivariable analysis, VAC, LVEF and Ea were found to be independent predictors of CI responders (as shown in Table 4). The logistic regression model was able to accurately classify 81.5% of CI responders, with an AUC of 0.943 (95% confidence interval 0.875–0.981, *P* < 0.001). The sensitivity was 77.8%, and the specificity was 85.1%. The positive and negative predictive values were 83.3% and 80.0%, respectively. Furthermore, every 0.1 increase in VAC was associated with a 5.534-fold rise in the likelihood of CI response.

**Table 4:** Multivariable logistic regressions with characteristics and haemodynamic covariates associated with cardiac index increase

	OR	95% confidence interval	*P*-Value
LVEF	0.891	0.816–0.972	0.010
SVRI	1.000	0.998–1.002	0.762
SVI	0.922	0.713–1.192	0.535
Ea × 10	3.035	1.459–6.310	0.003
VAC × 10	5.534	2.339–13.090	<0.001

Ea: arterial elastance index; LVEF: left ventricular ejection fraction; OR: odds ratio; SVI: stroke volume index; SVRI: systemic vascular resistance index; VAC: ventriculo-arterial coupling.

### Correlation between increasing cardiac index and the haemodynamic parameters

**Figure 2:**
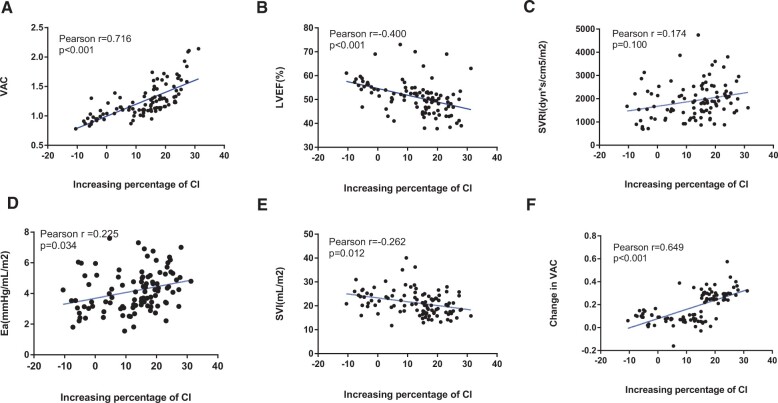
Correlation between increasing percentage of CI and pre-milrinone haemodynamic parameters. (**A**) VAC; (**B**) LVEF; (**C**) SVRI; (**D**) Ea; (**E**) SVI; and (**F**) change in VAC. We observed a significant correlation between the increasing percentage of CI and the pre-milrinone VAC. CI: cardiac index; Ea: arterial elastance; LVEF: left ventricular ejection fraction; SVI: stroke volume index; SVRI: systemic vascular resistance index; VAC: ventriculo-arterial coupling.

The results in Fig. 2 reveal significant positive correlations between pre-milrinone VAC, SVRI and Ea, and the percentage increase in CI through Parametric Pearson correlation analysis. In addition, LVEF and SVI prior to milrinone infusion were significantly negatively correlated with the rise in CI. Notably, VAC demonstrated the strongest correlation coefficient (*r* = 0.716, *P* < 0.001), while the correlations for the other variables were relatively weak.

### Predictability of ventriculo-arterial coupling, left ventricular ejection fraction and arterial elastance to distinguish cardiac index responders with milrinone

**Figure 3:**
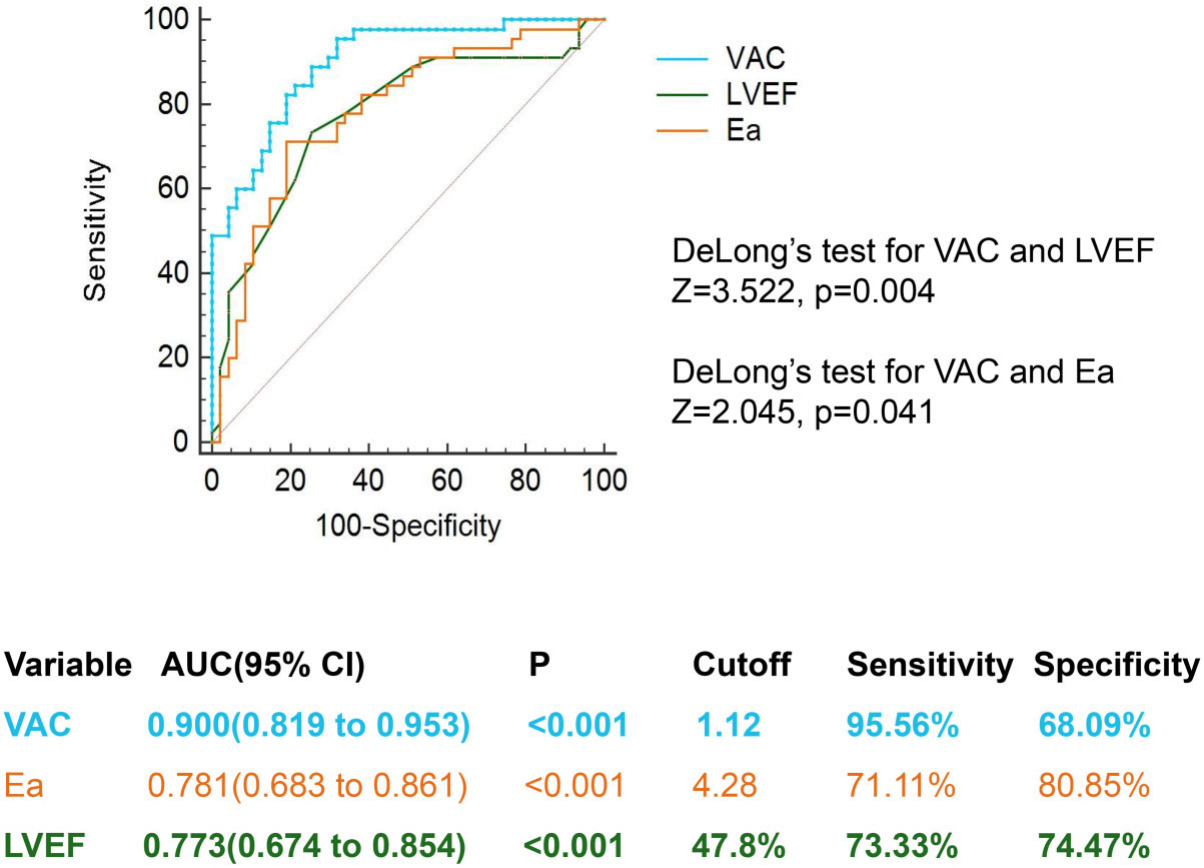
Receiver operating characteristic curves were calculated. DeLong's method was used to compare the values of the area under the curve (AUC). The highest AUC was achieved by pre-milrinone VAC (AUC = 0.900), followed by Ea (AUC = 0.781, *P* = 0.004) and LVEF (AUC = 0.773, *P* = 0.041). Ea: arterial elastance index; LVEF: left ventricular ejection fraction; VAC: ventriculo-arterial coupling.

The ROC curve analysis for pre-milrinone VAC to predict patients' CI responses revealed an AUC of 0.900 (95% confidence interval, 0.819–0.953, *P* < 0.0001) (Fig. 3). The cut-off value was 1.12, with a sensitivity of 95.56% and a specificity of 68.09%. DeLong's test demonstrated that the AUC of VAC was significantly greater than that of LVEF (*Z* = 3.522, *P* = 0.004) and Ea (*Z* = 2.045, *P* = 0.041).

## DISCUSSION

Our study found that the majority of CI responders exhibited high VAC after undergoing cardiac surgery, which was largely attributed to elevated Ea. Pre-milrinone VAC, Ea and LVEF all demonstrated predictive value for CI responders. However, pre-milrinone VAC exhibited the highest ROC AUC, surpassing Ea and LVEF. Therefore, pre-milrinone VAC could serve as an effective predictor of CI improvement following milrinone infusion.

Cardiology has solid evidence that VAC independently predicts treatment response [14, 15]. The results of our study confirmed the independent predictive value of VAC and further established VAC as a valuable tool in the field of paediatric heart surgery.

General monitoring, such as blood pressure, heart rate and CVP, cannot be relied on as sensitive indicators for detecting low cardiac output state. We did not observe any differences in the general monitoring variables between CI responders and non-responders. The variables of pre-milrinone LVEF, SVRI and SVI exhibited significant differences between the 2 groups. In CI responders, there was a significant increase in LVEF following milrinone infusion. However, it is difficult to differentiate whether the increased LVEF was due to decreased SVRI or increased myocardial contractility, as a decrease in SVRI may also be linked with an increase in LVEF [16]. Therefore, these variables alone may not provide a comprehensive explanation for the distinctions between CI responders and non-responders.

VAC, which is the ratio of Ea/Ees, integrates heart and vascular circulation. It accurately reflects the cohesive physiology of the cardiovascular system, where the cardiac and vascular systems should be matched [17, 18]. Their interaction may help elucidate the mechanisms underlying CI responsiveness. Ea is an integrative measure of arterial system properties, while Ees represents ventricular contractility.

After milrinone infusion, the change in Ea was significantly different between the 2 groups. In CI responders, there was a significant decrease in Ea, while no such change was observed in CI non-responders. This suggests that the difference in pre-milrinone vasomotor tone could be a possible explanation for the variation in response. As CI non-responders had low levels of Ea prior to milrinone infusion, there was no noticeable change in Ea.

Previous studies have indicated that the infusion of milrinone could enhance myocardial contractility [19]. However, our findings did not confirm the effectiveness of milrinone in enhancing myocardial contractility. One possible explanation is that there may be a natural decline in myocardial contractility during the early stage following cardiac surgery [20, 21]. Our findings suggest that the potential benefits of milrinone in enhancing cardiac contractility may be offset by the intrinsic decline in contractility that occurs at the early stage after cardiac surgery, resulting in no significant change in Ees. Generally, an increased afterload is coupled with a corresponding increase in left ventricular contractility. However, due to impaired myocardial function, Ees was unable to compensate for the elevation in Ea after CPB, resulting in low cardiac output and ventriculo-arterial decoupling.

The ratio of Ea/Ees serves as a measure of VAC, with a reference range of 0.7–0.8 for children [22]. A VAC ratio above 1.0 indicates ventricular arterial uncoupling. Our study revealed a relatively high VAC ratio above 1.0 following cardiac surgery, which was consistent with previous research [1, 4].

In CI responders, high pre-milrinone VAC was attributable to elevated Ea. Previous research has demonstrated that a high postoperative VAC was linked to poorer outcomes [4]. In our study, we found that a high pre-milrinone VAC was significantly associated with an increase in CI following milrinone infusion. Therefore, it is reasonable to speculate that optimizing VAC could lead to improved clinical outcomes.

In our study, the change in CI after milrinone infusion among CI responders may not be attributed to preload changes, as indicators of preload, such as inferior vena cava diameter/abdominal aorta diameter ratio and CVP, did not demonstrate significant changes following milrinone infusion.

Of all the variables examined, including pre-milrinone LVEF, SVRI, SVI, Ea and VAC, pre-milrinone VAC had the highest correlation coefficient with the increase in CI. This suggests that a high pre-milrinone VAC is a strong indicator of CI response following milrinone infusion. Our findings suggest that VAC may be a more reliable predictor of CI response in infants after cardiac surgery compared to LVEF and Ea, which separately reflect afterload and myocardial contractility.

Our study may have some important clinical implications. First, milrinone is often prescribed based on a physician's clinical experience to date. Our results suggest that VAC may serve as a clinical consideration for the use of milrinone, as it incorporates an integrated analysis of the cardiac and vascular state. Our findings indicate that infants with high VAC after CPB may be CI responders after milrinone infusion. Second, our findings highlight the possibility of personalized vasoactive agent treatment options based on VAC, which is a non-invasive echocardiographic assessment. Our results underscore the importance of considering VAC during the post-cardiac surgery period.

### Limitations

Several limitations of our study should be considered. First, the time interval between the pre- and post-administration of milrinone is 18–24 h, which is a considerable length of time, and as a result, the CI may spontaneously change over time. However, previous studies have indicated that instances of low cardiac output after congenital heart surgery typically occurred within the first 6–18 h [23]. Therefore, It is reasonable to deduce that the improvement of CI is relevant to milrinone. We also recognize that our data cannot prove the relationship between time and CI improvement. In our future study, we will research further to better gauge the impact of the time factor. Second, age can influence Ea and Ees, similar to other parameters such as systolic blood pressure, stroke volume and cardiac output, owing to physiological changes in the cardiovascular system [24]. Age-related effects can be partially controlled by indexing Ea and Ees against BSA, although this method is not commonly described. However, our primary focus was on the correlation between VAC and the increase in CI, as VAC is not influenced by age. Finally, this study has a small sample size. Therefore, further large-scale studies are needed to validate our findings, particularly in patients with high VAC after CPB.

## CONCLUSIONS

In infants with CHD after surgery, the change in CI following milrinone infusion depends on the VAC before milrinone administration. Infants with a pre-milrinone VAC of >1.12 can predict an increase in CI after milrinone infusion.

## Supplementary Material

ivad064_Supplementary_Data

## Data Availability

All relevant data are within the manuscript and its supporting Information files.

## ABBREVIATIONS

AUC: Area under the curve
BSA: Body surface area
CHD: Congenital heart disease
CI: Cardiac index
CPB: Cardiopulmonary bypass
CVP: Central venous pressure
Ea: Arterial elastance
Ees: Left ventricular end-systolic elastance
LCOS: Low cardiac output syndrome
LVEF: Left ventricular ejection fraction
OR: Odds ratio
ROC: Receiver operating characteristic
SVI: Stroke volume index
SVRI: Systemic vascular resistance index
VAC: Ventriculo-arterial coupling
