# Supplementary material for: Ventriculo-arterial coupling for predicting cardiac index increase in infants after heart surgery
Source: Interdiscip Cardiovasc Thorac Surg. 2023 May 9;37(3):ivad064. doi: 10.1093/icvts/ivad064 (PMC10497444; doi:10.1093/icvts/ivad064)
Supplement: ivad064_Supplementary_Data [file ivad064_supplementary_data.zip › supple 1.doc]

**Supplemental 1**

The calculation formulars of hemodynamic variables are as follows:

LV stroke volume (SV, ml) =Diameter（LVOT）^2^*0.785

Stroke volume index (SVI, ml/m^2^) =SV/ body surface area;

Cardiac index (CI; l/min/m^2^) = HR*SVI/1000;

Systemic vascular resistance index (SVRI, dyn*s/cm^5^/m^2^) =80*(MBP-CVP)/CI

Abbreviations:

SV=stroke volume;

LVOT=left ventricular outflow tract;

SVI=stroke volume index;

CI=cardiac index;

HR=heart rate;

SVRI=systemic vascular resistance index;

MAP= mean blood pressure;

CVP=central venous pressure.
