# Supplementary material for: Ventriculo-arterial coupling for predicting cardiac index increase in infants after heart surgery
Source: Interdiscip Cardiovasc Thorac Surg. 2023 May 9;37(3):ivad064. doi: 10.1093/icvts/ivad064 (PMC10497444; doi:10.1093/icvts/ivad064)
Supplement: ivad064_Supplementary_Data [file ivad064_supplementary_data.zip › Supple 3.doc]

Supplemental 3. Comparison of hemodynamic variables before and 24hours after milrinone infusion

| **Hemodynamic variables** | **pre milrinone** | **post milrinone** | **p** |
| --- | --- | --- | --- |
| **HR (bpm)** |  |  |  |
| All (n=92) | 137(21) | 136(26) | 0.390 |
| CI responders (n=45) | 142(22) | 133(25) | 0.073 |
| CI nonresponders (n=47) | 132(26) | 137(30) | 0.453 |
| **SBP (mmHg）** |  |  |  |
| All (n=92) | 94(21) | 91(24) | 0.368 |
| CI responders (n=45) | 95(21) | 90(28) | 0.341 |
| CI nonresponders (n=47) | 93(22) | 94(21) | 0.822 |
| **MBP (mmHg）** |  |  |  |
| All (n=92) | 73(21) | 70(24) | 0.368 |
| CI responders (n=45) | 74(20) | 70(26) | 0.416 |
| CI nonresponders (n=47) | 73(22) | 71(21) | 0.653 |
| **CVP （mmHg)** |  |  |  |
| All (n=92) | 7.7(3.1) | 8.0(2.9) | 0.473 |
| CI responders (n=45) | 7.7(3.3) | 8.0(2.8) | 0.630 |
| CI nonresponders (n=47) | 7.7(3.2) | 7.9(3.0) | 0.752 |
| **IVC:AA** |  |  |  |
| All (n=92) | 1.19(0.20) | 1.21(0.26) | 0.559 |
| CI responders (n=45) | 1.15(0.20) | 1.20(0.25) | 0.298 |
| CI nonresponders (n=47) | 1.20(0.19) | 1.23(0.27) | 0.535 |
| **LVEF (%)** |  |  |  |
| All (n=92) | 51.8(6.5) | 55.2(5.3) | <0.001 |
| CI responders (n=45) | 47.5(7.1) | 55.0(5.5) | <0.001 |
| CI nonresponders (n=47) | 53.4(7.5)^A^ | 55.9(5.2) | 0.063 |
| **SVRI (dynes /sec /cm-^5^ / m^2^)** |  |  |  |
| All (n=92) | 1931(774) | 1599(536) | <0.001 |
| CI responders (n=45) | 2064(784) | 1520(472) | <0.001 |
| CI nonresponders (n=47) | 1720(528)^A^ | 1616(672) | 0.407 |
| **SVI (ml/m^2^)** |  |  |  |
| All (n=92) | 21.4(5.1) | 23.2(6.2) | 0.033 |
| CI responders (n=45) | 18.8(3.5) | 22.4(4.1) | <0.001 |
| CI nonresponders (n=47) | 23.6(4.9)^A^ | 23.2(7.1) | 0.751 |
| **CI (L/min/m^2^)** |  |  |  |
| All (n=92) | 2.89(0.52) | 3.22(0.53) | <0.0001 |
| CI responders (n=45) | 2.63(0.35) | 3.17(0.45) | <0.0001 |
| CI nonresponders (n=47) | 3.14(0.56)^A^ | 3.24(0.66) | 0.430 |
| **Pre ejction time(ms)** |  |  |  |
| All (n=92) | 57(16) | 53(17) | 0.102 |
| CI responders (n=45) | 58(17) | 53(19) | 0.192 |
| CI nonresponders (n=47) | 55(16) | 54(15) | 0.755 |
| **Total ejection time(ms)** |  |  |  |
| All (n=92) | 222(29) | 219(29) | 0.484 |
| CI responders (n=45) | 221(29) | 215(31) | 0.346 |
| CI nonresponders (n=47) | 223(29) | 225(28) | 0.735 |
| **Ea (mmHg/ml/m^2^)** |  |  |  |
| All (n=92) | 4.17(1.32) | 3.51(0.92) | <0.001 |
| CI responders (n=45) | 4.70(1.15) | 3.47(0.74) | <0.001 |
| CI nonresponders (n=47) | 3.66(0.84)^A^ | 3.54(1.03) | 0.537 |
| **Ees(mmHg/ml/m^2^)** |  |  |  |
| All (n=92) | 3.46(1.05) | 3.47(0.51) | 0.682 |
| CI responders (n=45) | 3.45(1.15) | 3.43(0.34) | 0.911 |
| CI nonresponders (n=47) | 3.47(0.87) | 3.50(0.54) | 0.841 |
| **VAC** |  |  |  |
| All (n=92) | 1.25(0.28) | 1.00(0.18) | <0.001 |
| CI responders(n=45) | 1.43(0.29) | 1.01(0.20) | <0.001 |
| CI nonresponders(n=47) | 1.07(0.17)^A^ | 1.00(0.18) | 0.059 |
| **VIS** |  |  |  |
| All (n=92) | 5.5(4.8) | 10.5(4.8) | <0.001 |
| CI responders (n=45) | 5.4(5.1) | 10.2(5.4) | <0.001 |
| CI nonresponders (n=47) | 4.8(4.9) | 11.0(5.1) | <0.001 |

HR= heart rate, SBP=systolic blood pressure, MBP=mean blood pressure, CVP=central venous pressure, IVC:AA=the inferior vena cava to abdominal aorta diameter index, LVEF=Left ventricular ejection fraction, SVRI=systemic vascular resistance index, SVI=stroke volume index, CI=cardiac index, Ea = arterial elastance, Ees = end-systolic ventricular elastance, VAC=ventriculo-arterial coupling, VIS=Vasoactive-inotropic score

^A^ p < 0.05 refers to comparison between cardiac index (CI) responders and CI nonresponders.

P-value refers to the comparison between post- and pre- milrinone.
