# Supplementary material for: Ventriculo-arterial coupling for predicting cardiac index increase in infants after heart surgery
Source: Interdiscip Cardiovasc Thorac Surg. 2023 May 9;37(3):ivad064. doi: 10.1093/icvts/ivad064 (PMC10497444; doi:10.1093/icvts/ivad064)
Supplement: ivad064_Supplementary_Data [file ivad064_supplementary_data.zip › Supplemental 2.doc]

Supplemental 2. Diagnosis of study population

| Diagnosis | N (%) |
| --- | --- |
| Patent ductus arteriosus surgery at age | 8 (8.70%) |
| Ventricular septal defect | 13 (14.13%) |
| Atrial septal defect | 4(4.35%) |
| Tetralogy of Fallot | 7(7.61%) |
| Atrioventricular septal defect | 4(4.35%) |
| Aorta-pulmonary window | 2 (2.17%) |
| Ebstein anomaly | 1(1.09%) |
| Pulmonary stenosis | 7 (7.61%) |
| Total anomalous pulmonary venous connection | 6 (6.52%) |
| Anomalous left coronary artery from the pulmonary artery | 3 (3.26%) |
| Pulmonary atresia | 14(15.22%) |
| Double outlet right ventricle | 8(8.70%) |
| Interrupted aortic arch | 3 (3.26%) |
| Transposition of the great arteries | 3 (3.26%) |
| Coarctation and ventricular septal defect closure | 3 (3.26%) |
| Single ventricle | 2(2.17%) |
| Truncus arteriosus | 1(1.09%) |
| Interrupted aortic arch with ventricular septal defect closure | 1(1.09%) |
| Truncus arteriosus and interrupted arch | 1(1.09%) |
| Other types of CHD | 1(1.09%) |
